# Supplementary material for: Ezrin and Its Phosphorylated Thr567 Form Are Key Regulators of Human Extravillous Trophoblast Motility and Invasion
Source: Cells. 2023 Feb 23;12(5):711. doi: 10.3390/cells12050711 (PMC10000480; doi:10.3390/cells12050711)

Ezrin and its phosphorylated Thr567 form are key regulators of human extravillous trophoblast motility and invasion

Maral E. A. Tabrizi, Janesh Gupta and Stephane R. Gross

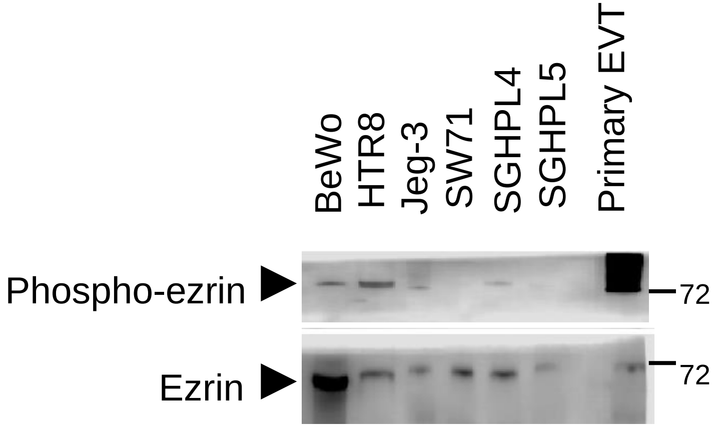

Supplementary Figure S1: Ezrin is highly phosphorylated in primary EVTs compared to all trophoblast cell lines. Choriocarcinoma cells Bewo and Jeg-3 cells, along with EVT cell lines HTR8/SVneo, Swan71, SGHPL4 and SGHPL5 as well as primary EVT cells were grown for 48 hours prior to collection of the protein lysates for Western blotting. Cells were collected and solubilised in Lammeli buffer and equal loading were separated by SDS-PAGE electrophoresis. Western blotting was carried out and membranes probed for ezrin and Thr567 phospho-ezrin. Cropped images are presented.

Ezrin and its phosphorylated Thr567 form are key regulators of human extravillous trophoblast motility and invasion

Maral E. A. Tabrizi, Janesh Gupta and Stephane R. Gross

Supplementary Figures S2: Original blots for all Western blot analysis presented in the manuscript.

Refer to figure legends in the manuscript for all conditions and setup followed for any specific experiments.

In general, either human tissues or cells (Choriocarcinoma cells Bewo and Jeg-3 cells, along with EVT cell lines HTR8/SVneo, Swan71, SGHPL4 and SGHPL5 as well as primary EVT cells) were prepared as protein lysates in Lammeli buffer for Western blotting. Equal loading were separated by SDS-PAGE electrophoresis. Western blotting was carried out and membranes probed for ezrin, α-tubulin and Thr567 phospho-ezrin.

Article

Ezrin and its phosphorylated Thr567 form are key regulators of human extravillous trophoblast motility and invasion

Maral E. A. Tabrizi, Janesh Gupta and Stephane R. Gross

Supplementary Figures S2

Original blot images from Figure 1

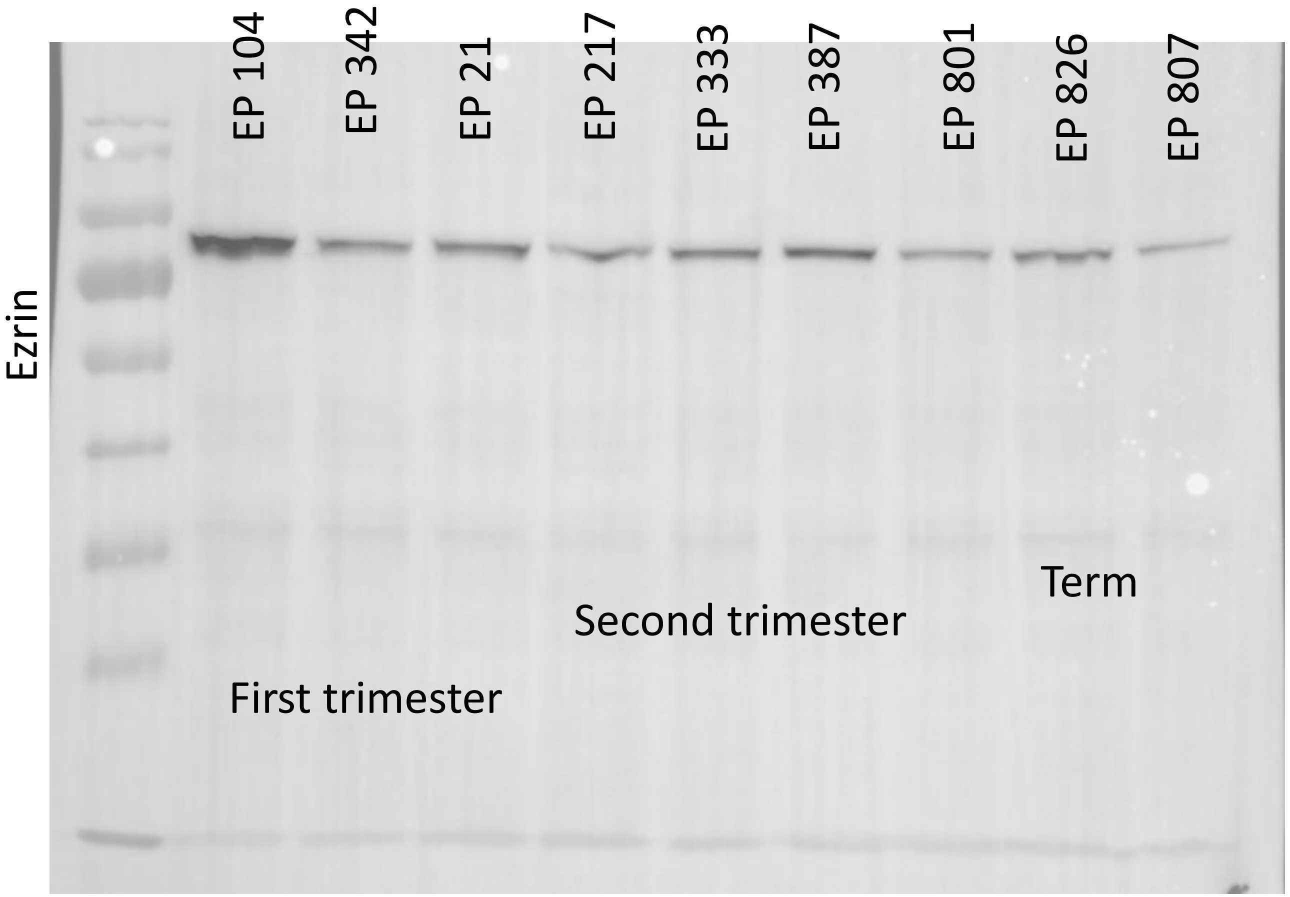

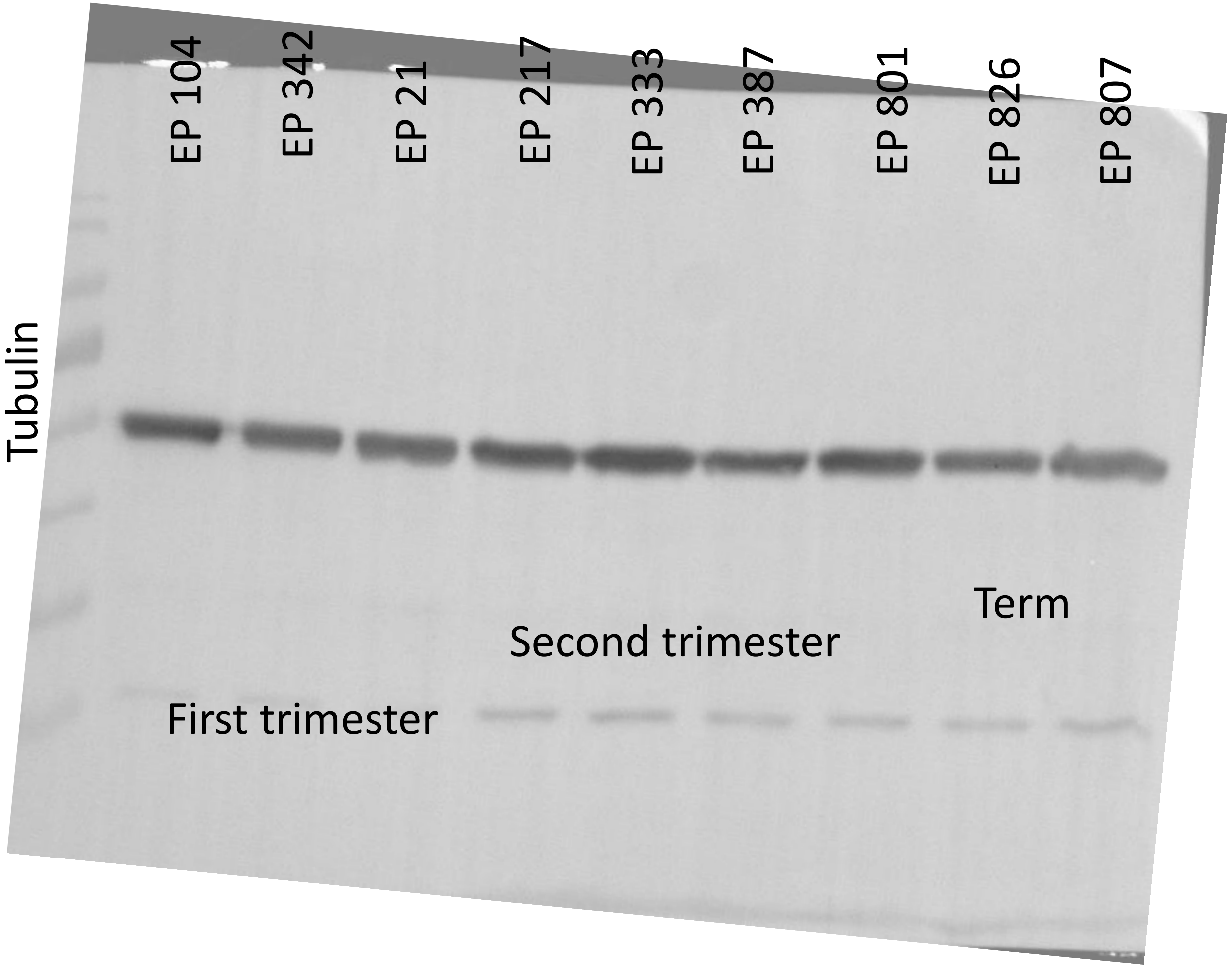

Original blot images from Figure 2

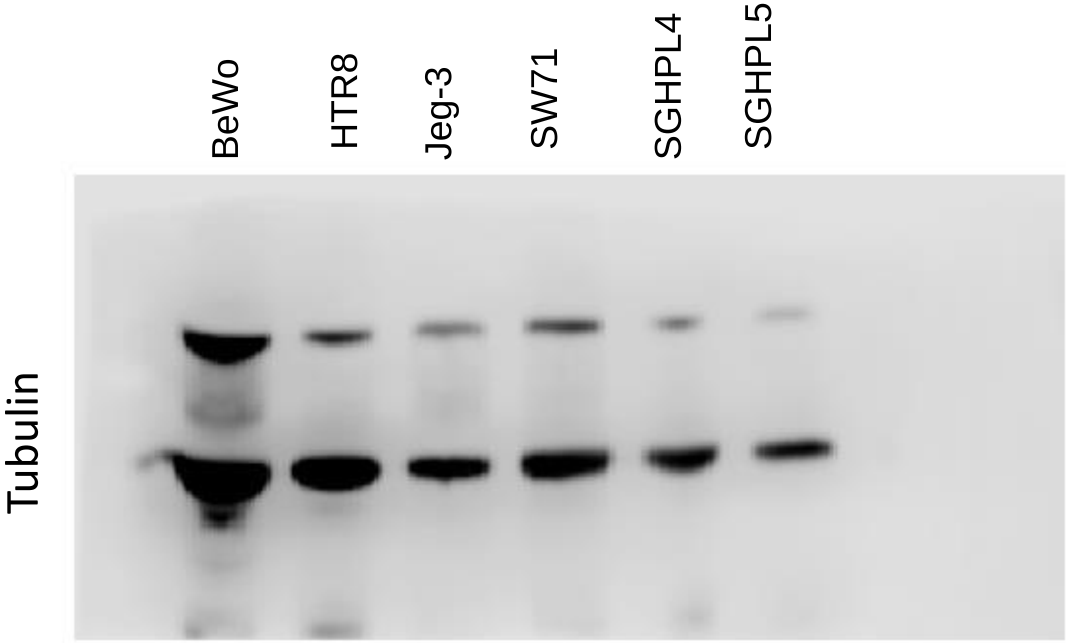

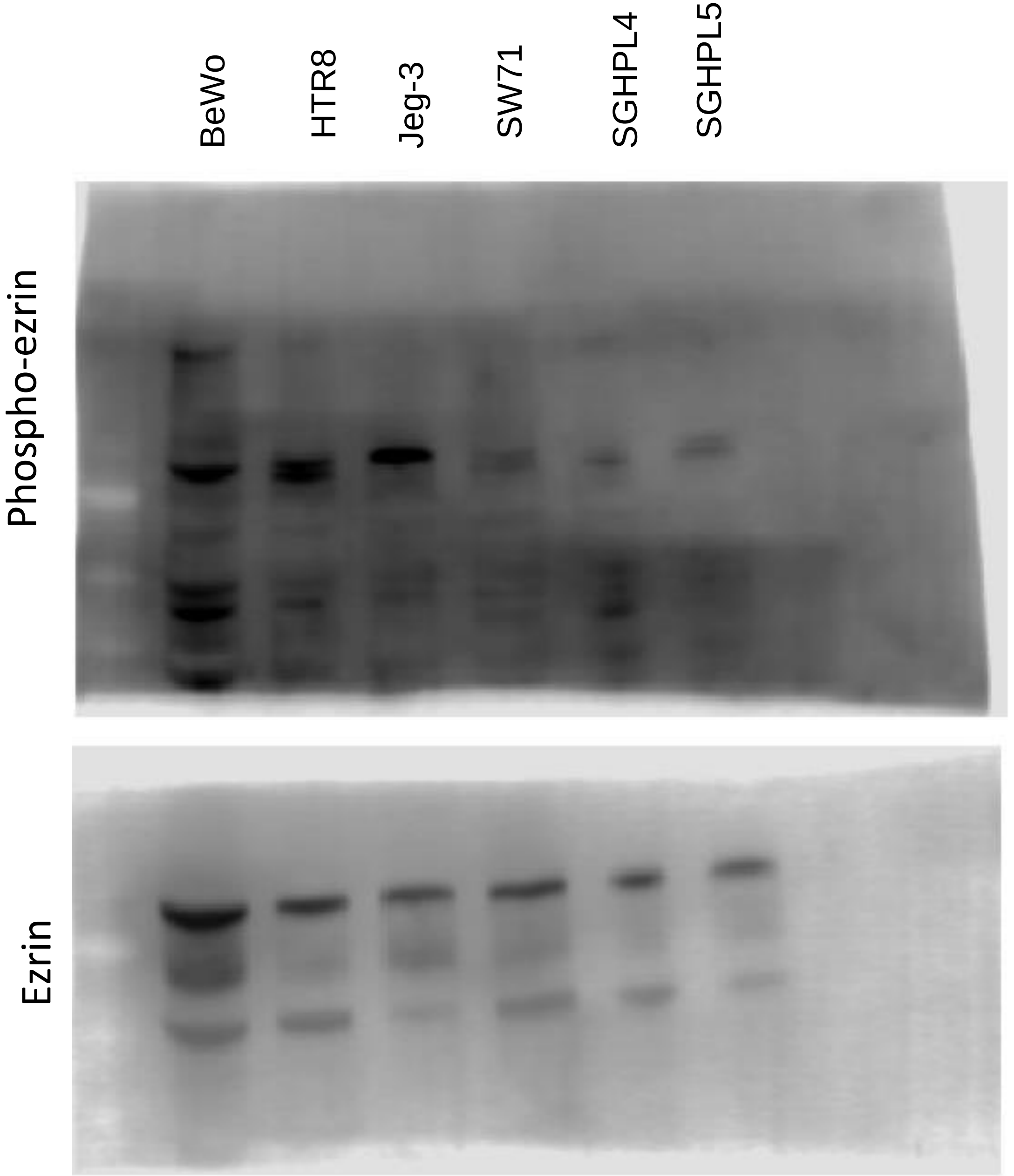

Original blot images from Figure 3: siRNA treatment

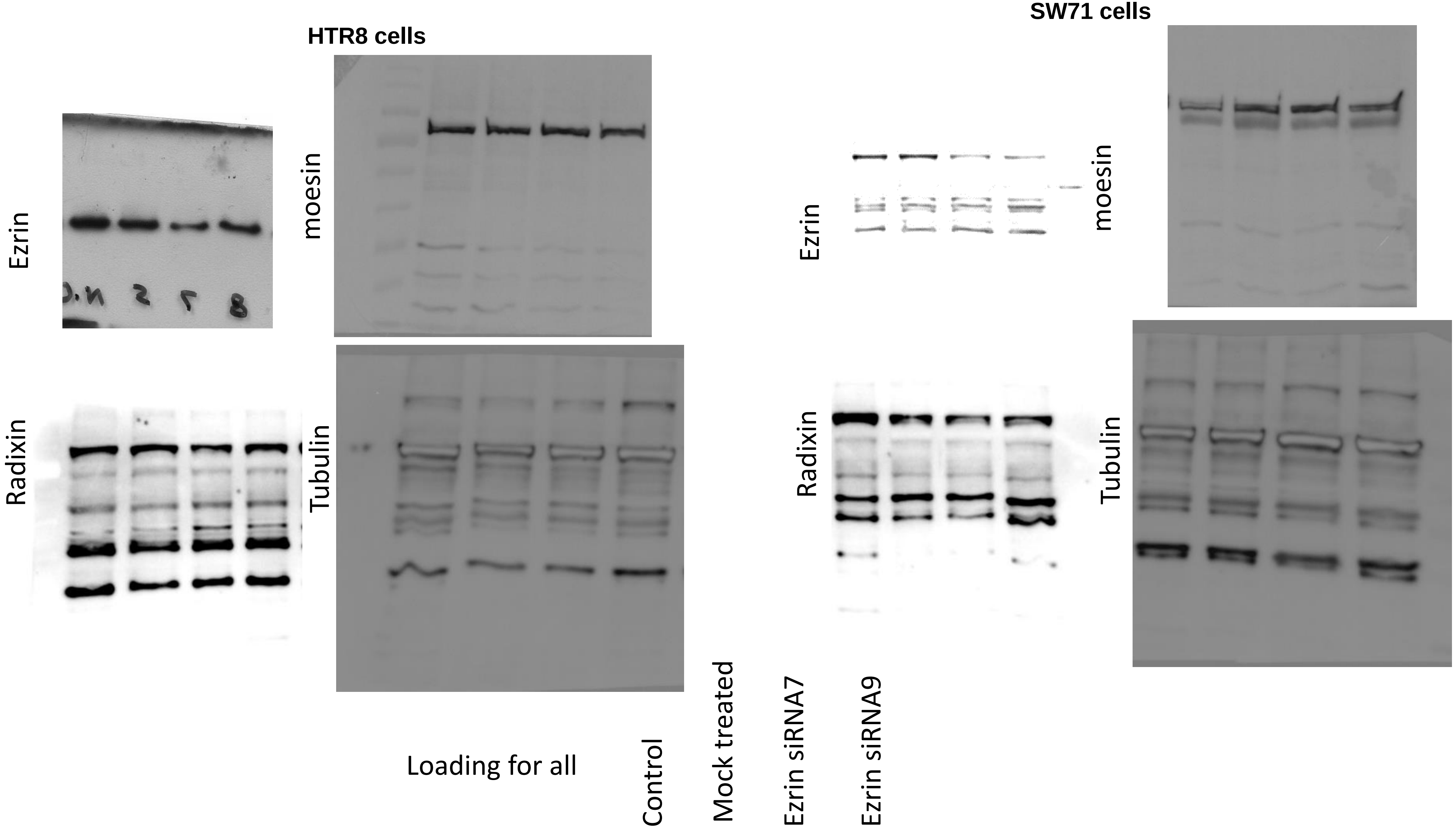

Original blot images from Figure 3: NCS668394 treatment

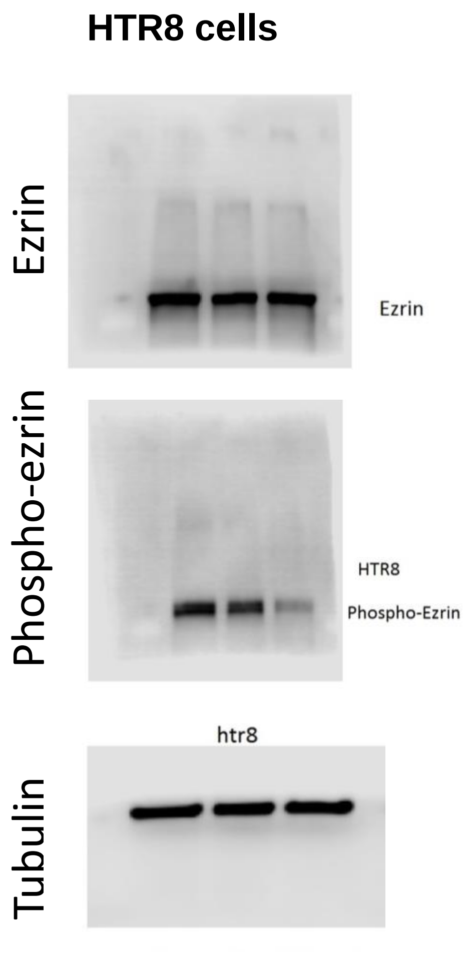

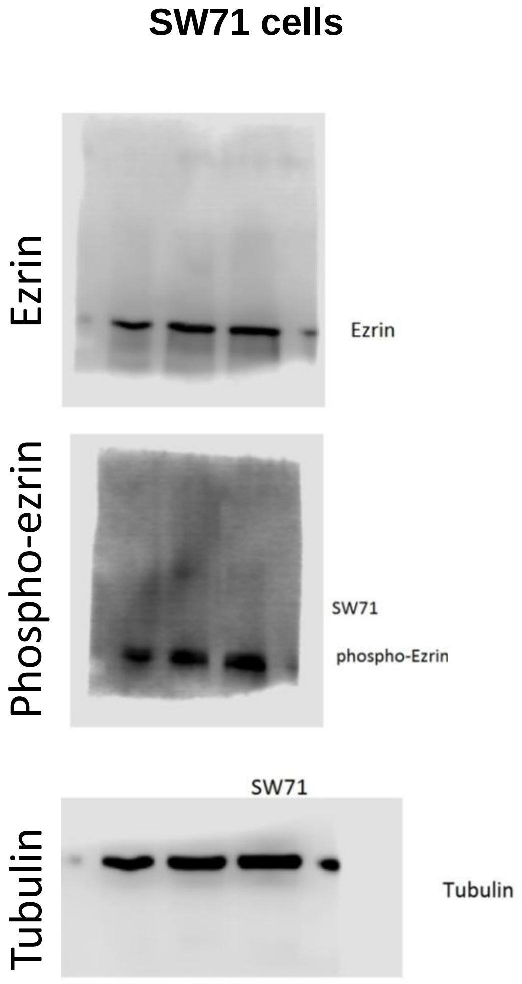

Loading for all
Control
DMSO
NCS668394

Original blot images from Figure 7

Ezrin

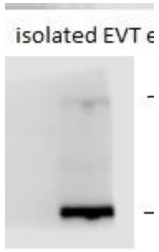

Phospho-ezrin

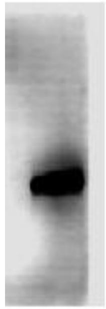

EVTs E

Tubulin

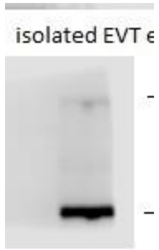

Original blot images from Figure S1 Phospho-ezrin in all trophoblast cells lines and Primary EVTs

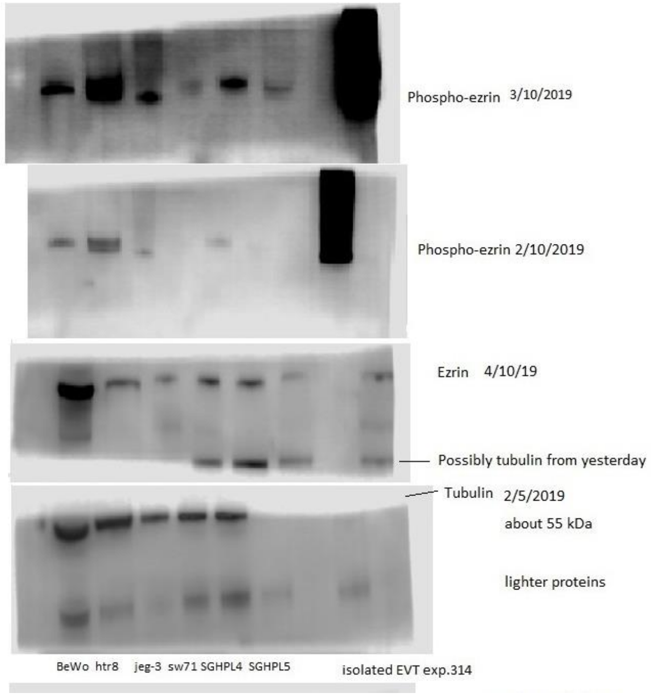

Supplement: Supplementary file 1 [file cells-12-00711-s001.zip › cells-2177434-supplementary.pdf]
