# Supplementary material for: Ezrin and Its Phosphorylated Thr567 Form Are Key Regulators of Human Extravillous Trophoblast Motility and Invasion
Source: Cells. 2023 Feb 23;12(5):711. doi: 10.3390/cells12050711 (PMC10000480; doi:10.3390/cells12050711)
Supplement: Supplementary file 1 [file cells-12-00711-s001.zip › Supplementary Tables .pdf]

Supplementary Tables

Table S1: Antibodies used in this study.

Antibody	Experiment	Type	Clone	Supplier	Dilution	Incubation	Buffer
**Ezrin**	IHC	Monoclonal	IgG Mouse	Abcam	1/200	Overnight at 4⁰C	2.5% goat serum
**HLA-G**	IHC	Monoclonal	IgG1 Mouse	Abcam	1/100	Overnight at 4⁰C	2.5% goat serum
**Cytokeratin 7**	IHC	Monoclonal	IgG Mouse	Abcam	1/100	Overnight at 4⁰C	2.5% goat serum
**Ezrin**	IF	Monoclonal	IgG Mouse	Abcam	1/200	Overnight at 4⁰C	2.5% goat serum
**Phospho-ezrin**	IF	Monoclonal	IgG Mouse	Abcam	1/200	Overnight at 4⁰C	2.5% goat serum
**Paxillin**	IF	Monoclonal	Mouse	Invitrogen	1/200	45 minutes at room temperature	1% Goat serum
**α−Tubulin**	WB	Monoclonal	Mouse	Sigma	1/5000	Overnight at 4⁰C	3% BSA
**Ezrin**	WB	Monoclonal	IgG Mouse	Abcam	1/1000	Overnight at 4⁰C	3% BSA
**Phospho-ezrin**	WB	Monoclonal	IgG Mouse	Abcam	1/1000	Overnight at 4⁰C	3% BSA
**Radixin**	WB	Monoclonal	IgG Rabbit	Insight Biotech	1/1000	Overnight at 4⁰C	3% BSA
**Moesin**	WB	Monoclonal	IgG Rabbit	Insight Biotech	1/1000	Overnight at 4⁰C	3% BSA
**Mouse**	WB	Biotinylated	IgG Horse Anti-Mouse				
**Rabbit**	WB	Biotinylated	IgG Goat Anti-Rabbit				
**Rabbit IgG (negative control)**	IHC			Santa Cruz	1/2000	Overnight at 4⁰C	2.5% goat serum
**Mouse IgG (negative control)**	IHC			Santa Cruz	1/100	Overnight at 4⁰C	2.5% goat serum

Table S2: Total number of cells counted for motility and invasion of HTR8/SVneo and Swan71 trophoblast cell lines and primary EVT cells after ezrin knock down or NSC668394 treatment.

Cell lines	Control	Mock treatment	Ezrin siRNA 7 treatment	Ezrin siRNA 9 treatment	NSC668394 treatment
**HTR8/SVneo motility**	2724	2884	2321	1032	207
**HTR8/SVneo invasion**	2541	2349	1505	1079	91
**Swan71 motility**	7436	2388	1440	957	2386
**Swan71 invasion**	1731	814	228	157	331
**Primary EVT motility**	1349	N/A	N/A	N/A	740
**Primary EVT invasion**	1077	N/A	N/A	N/A	757

HTR8/SVneo cells and Swan71 cells as well as primary first trimester EVTs cells were either left untreated, mock treated or incubated with either ezrin siRNA7, ezrin siRNA9 or NSC668394 prior to seeding on Boyden chambers for motility or matrigel-coated Boyden chambers for invasion. Data presented in this Table are the sum of all cells counted on the far side of the chambers at the end of the incubation from three or four independent experiments (Figures 4, 5 and 7).
